# Supplementary material for: Genomic Characterization and Wetland Occurrence of a Novel Campylobacter Isolate from Canada Geese
Source: Microorganisms. 2023 Mar 3;11(3):648. doi: 10.3390/microorganisms11030648 (PMC10056850; doi:10.3390/microorganisms11030648)
Supplement: Supplementary file 1 [file microorganisms-11-00648-s001.zip › microorganisms-2208937-supplementary.pdf]

SUPPLEMENTARY DATA

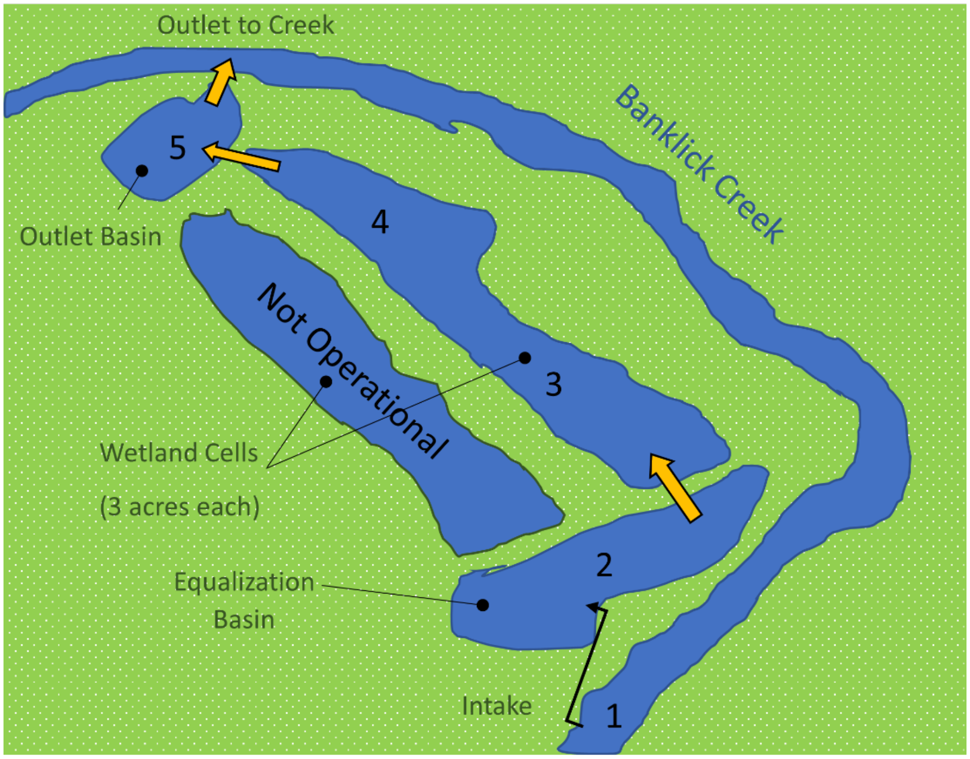

Supplementary Figure S1. Banklick Creek wetland site overview with sampling locations 1 through 5. Figure is based on (McMinn et al., 2019).

```
CP012543.1 Campylobacter rectus        ACCAACTAGCTGATACGATATAGCCTCATCCTACACCGAAAAACTTTCCCGACTTAACTT
CP046243.1 Campylobacter lari          ACCAACTAGCTGATACTATATAGTCTCATCCTACACCGAAAAACTTTCCCTACTCAACTT
CP083814.1 Campylobacter coli          ACCAACTAGCTGATACTATATAGTCTCATCCTACACCGAAAAACTTTCCCTACTCAACTT
CP048760.1 Campylobacter jejuni        ACCAACTAGCTGATACTATATAGTCTCATCCTACACCGAAAAACTTTCCCTACTCAACTT
CP035946.1 Campylobacter canadensis    ACCAACTAGCTGATACAATATAGCCCTATCCCTTACCGAAAAACTTTCCCACCTTAACTT
MG1                                    ACCAACTAGCTGATACAATATAGCCCTATCCCTTACCGAAAAACTTTCCCACCCTAACTT
                                       **************** ****** *  ****   ****************  *  *****

CP012543.1 Campylobacter rectus        ATGTAAAGAAGGAGTATAGAGTATTAGCAGTCGTTTCCAACTGTTGTCCTCTAGTGTAGG
CP046243.1 Campylobacter lari          ATGTTAAGCAGGAGTATAGAGTATTAGCAGTCGTTTCCAACTGTTGTCCTCTTGTGTAGG
CP083814.1 Campylobacter coli          GTGTTAAGCAGGAGTATAGAGTATTAGCAGTCGTTTCCAACTGTTGTCCTCTTGTGTAGG
CP048760.1 Campylobacter jejuni        GTGTTAAGCAGGAGTATAGAGTATTAGCAGTCGTTTCCAACTGTTGTCCTCTTGTGTAGG
CP035946.1 Campylobacter canadensis    ATGTTAAAGCGGAGTATAGAGTATTAGCAGCCATTTCTAGCTGTTGTCCTCTTGTAAGGG
MG1                                    ATGTTAGAGCGGAGTATAGAGTATTAGCACTCATTTCTAAGTGTTGTCCTCTTGTAAGGG
                                        *** *    *******************  * **** *  *********** **   **

CP012543.1 Campylobacter rectus        GCAGATTAGCTATATATTACTCACCCGTGCGCCACT
CP046243.1 Campylobacter lari          GCAGATTAACTATACCTTACTCACCCGTGCGCCACT
CP083814.1 Campylobacter coli          GCAGATTAACTATACCTTACTCACCCGTGCGCCACT
CP048760.1 Campylobacter jejuni        GCAGATTAACTATACCTTACTCACCCGTGCGCCACT
CP035946.1 Campylobacter canadensis    GCAAGTTAGCTATACATTACTCACCCGTGCGCCACT
MG1                                    GCAAGTTAGCTATACATTACTCACCCGTGCGCCACT
                                       ***  *** *****  ********************
```

- F Primer
- Probe
- R Primer
- Misalignments against MG1 in primer/probe regions

Supplementary Figure S2. Multiple sequence alignment of MG1 and five *Campylobacter* spp. used for design of real-time PCR primers. Colors indicate primer/probe regions (green, purple, blue) and regions containing MG1-unique nucleotides (red).

Supplementary Table S1. List of primers and probes used in PCR and qPCR.

Target	Purpose	Amplicon length	Name	Sequence (5'-3')	Reference
Campylobacter (genus)*	PCR	716bp	C412F<br>C2388R	GGATGACACTTTTCGGAGC<br>CATTGTAGCTCGTGTGTC	Linton et al. 1996
MG1	qPCR	102bp	MG16S_F1<br>MG16S_R3<br>MG16S_Probe	ACCGAAAAACTTTCCCACCCT<br>TGTATAGCTAACTTGCCCCTTAC<br>TAGAGCGGAGTATAGAGTATTAGCACTCA	n/a
Campylobacter (genus)	qPCR	108bp	Camp_F2<br>Camp_R2<br>Camp_P2	CACGTGCTACAATGGCATAT<br>GGCTTCATGCTCTCGAGTT<br>FAM-CAGAGAACAATCCGAACTGGGACA-TAMRA/BHQ	Lund et al. 2004
Campylobacter (genus)	Sanger Sequencing	905bp	C412F<br>Camp_R2	GGATGACACTTTTCGGAGC<br>GGCTTCATGCTCTCGAGTT	Linton et al. 1996<br>Lund et al. 2004

Supplementary Table S2. Specificity of MG1 qPCR primer and probe.

Campylobacter strain*	Host	Detection
C. jejuni C1	Human	n.d.
C. jejuni C2	Human	n.d.
C. jejuni C3	Human	n.d.
C. lari 58BB	Gull	n.d.
C. jejuni 63A	Gull	n.d.
C. lari 64BB	Gull	n.d.
C. volucri 70BB	Gull	n.d.
C. jejuni K1	Crow	n.d.
C. jejuni K2	Crow	n.d.
C. jejuni	Laboratory	n.d.
C. coli	Laboratory	n.d.
C. lari	Laboratory	n.d.
MG1	Canada Goose	+

*See Lu et al. 2019 for strain references
n.d. = not detected

Supplementary Table S3. Detection of MG1 in Canada goose fecal material surrounding Banklick Creek wetland site.

Sample	Date Collected	Quantification (copy/mL)
1	8/23/2017	3931
2	9/14/2017	8849
PC*	\	9.48E+07
NC*	\	n.d.

*Positive control (pure culture)
**Negative control
n.d. = not detected
